# Health anxiety and physical activity among young and middle-aged employees: the role of self-compassion and coping strategies

**DOI:** 10.3389/fpsyg.2025.1678651

**Published:** 2026-01-12

**Authors:** Ke Xu, Hongyu Jiang, Huilin Wang

**Affiliations:** 1 School of Physical Education, Hunan University of Science and Technology, Xiangtan, China; 2 School of Business, Hunan University of Science and Technology, Xiangtan, China

**Keywords:** health anxiety, self-compassion, coping strategies, physical activity, young and middle-aged employees

## Abstract

**Introduction:**

Health anxiety significantly affects young and middle-aged employees, leading to lower productivity, absenteeism, and even serious symptoms like depression or suicidal thoughts. Physical activity offers a win–win solution by relieving health anxiety and improving overall well-being, which can indirectly benefit both the economy and businesses. This study focuses on how to encourage physical activity among those experiencing health anxiety.

**Methods:**

Using a cross-sectional design, this study gathered data through a combination of snowball and convenience sampling. A total of 423 valid questionnaires were obtained from employees across the public sector, manufacturing, services, and IT industries in three Chinese provinces: Shandong, Jiangxi, and Anhui. The research model and hypotheses were evaluated using structural equation modeling with SmartPLS.

**Results:**

The results indicate that health anxiety among young and middle-aged employees is positively associated with self-compassion and coping strategies. In turn, self-compassion contributes positively to the adoption of effective coping strategies, and both factors are linked to higher levels of physical activity participation. Moreover, self-compassion and coping strategies jointly serve as mediating mechanisms through which health anxiety influences individuals’ engagement in physical activity.

**Discussion:**

Health anxiety can motivate young and middle-aged workers to adopt self-compassion and coping strategies, promoting physical activity. Governments and enterprises should improve sports facilities and foster active workplace cultures to support this. Meanwhile, employees are encouraged to choose physical activity as a healthy coping method to avoid the harms of maladaptive strategies.

## 1 Introduction

Health anxiety refers to a negative psychological condition characterized by excessive worry about the possibility of developing illness in the future. Individuals suffering from health anxiety tend to spend significant amounts of time seeking help from others or accessing healthcare resources for reassurance and treatment (Nagao et al., 2023). Early studies in specific regions have shown that over 90% of working individuals reported concerns about their health (Indregard et al., 2013). Since the COVID-19 pandemic, the incidence of illness and perceived health threats has increased, leading to a marked rise in health anxiety among the working population. This trend is particularly pronounced among young and middle-aged employees, who often lack experience in managing health-related concerns (Duplaga and Grysztar, 2021; Mokhtari et al., 2020), a pattern that appears especially salient in China, where strict control measures and prolonged disruptions may have amplified health-related worries.

Health anxiety is a key contributor to occupational stress and reduced self-efficacy, often leading to lower job satisfaction, absenteeism, and decreased productivity. It is also linked to psychological and physical symptoms such as depression, headaches, and sleep disturbances, and in severe cases, to social withdrawal, phobias, obsessive-compulsive behaviors, and suicidal thoughts (Mokhtari et al., 2020; Shan et al., 2022; Heinen et al., 2021). To cope, some individuals with health anxiety cope by excessively checking their bodies or searching online for information in an attempt to gain reassurance. Others may resort to maladaptive behaviors such as increased smoking or alcohol consumption (Pasha et al., 2022; Lizano, 2015; Tyrer, 2018). However, research Peng (2022) and Ferretti (2015) indicate that certain coping behaviors may, paradoxically, intensify anxiety levels and contribute to adverse health outcomes. These maladaptive strategies have been associated with elevated risks of developing serious physical conditions, including cardiovascular diseases, chronic respiratory disorders, and various forms of cancer.

In contrast, some individuals adopt healthier coping strategies, with physical activity widely recognized for its mental health benefits. Regular exercise can reduce anxiety and depression, boost self-confidence, and lower suicide risk (Doré et al., 2016), while also improving cardiovascular and respiratory health (Stubbs et al., 2017). Kandola et al. (2018) further revealed that physical activity promotes positive neurobiological changes in brain regions involved in anxiety regulation, thereby mitigating health anxiety symptoms. Moreover, because physical activity induces physiological responses (e.g., elevated heart rate) that resemble those triggered by anxiety, it may help individuals become less reactive to such sensations over time. Consequently, sustained engagement in physical activity may serve both as a preventive and therapeutic measure against health anxiety.

Existing studies highlight physical activity as a promising non-pharmacological intervention for health anxiety. Increased activity is generally linked to lower anxiety levels (Anderson and Shivakumar, 2013). McDowell et al. (2019) and Wang et al. (2023) found a significant inverse relationship between physical activity and health anxiety. Kirmizi et al. (2021) suggested that it may relieve musculoskeletal symptoms, while Carter et al. (2021) noted that enhanced self-efficacy from exercise helps reduce anxiety. At the same time, in studies based on the Chinese context, scholars have tended to emphasize how self-compassion and coping strategies alleviate individuals’ health anxiety. For example, a study of Chinese medical students found that self-compassion helps promote the use of healthier behaviors for self-regulation, thereby mitigating the negative impact of health anxiety (Lu et al., 2025). A study of Chinese university students reported that positive coping strategies can effectively enhance psychological resilience, which in turn reduces health anxiety (Yang et al., 2025). Despite these advances, little is known about how self-compassion and coping strategies jointly operate in relation to physical activity among working adults.

However, few studies have explored how self-compassion and coping strategies jointly mediate the association between health anxiety and physical activity, particularly among young and middle-aged employees. This population, especially in the post-COVID-19 era, has become increasingly attentive to their physical and mental well-being. When they struggle to adapt to bodily or emotional changes and misinterpret these sensations as signs of serious illness, anxiety may arise. In response, many may turn to adaptive behaviors, such as engaging in physical activity, as a way to manage their distress. Drawing on protection motivation theory and self-compassion theory, the present study focuses on working adults and examines how psychological processes related to threat appraisal and coping appraisal are associated with their physical activity behavior. Against this backdrop, this study aims to: (1) examine the relationships among health anxiety, self-compassion, coping strategies, and physical activity; (2) assess the mediating roles of self-compassion and coping strategies; and (3) provide practical recommendations to support mental health and encourage active lifestyles among young and middle-aged workers.

## 2 Literature review and hypothesis development

### 2.1 Theoretical framework

This study draws on protection motivation theory and self-compassion theory. Rogers (1975) proposed protection motivation theory, arguing that when individuals evaluate a potential event and judge that it may have negative consequences, they generate a protection motivation that leads them to consciously choose appropriate coping measures to prevent the occurrence of negative events. Within this framework, threat appraisal reflects individuals’ perceptions of the severity of and vulnerability to a health threat (Lek and Bishop, 1995), whereas coping appraisal concerns the evaluation of one’s ability to perform a recommended response (self-efficacy) and the perceived effectiveness and costs of that response (response efficacy and response costs) (Teasdale et al., 2012). In the present study, health anxiety is conceptualized as an outcome of heightened threat appraisal, capturing perceived susceptibility to and concern about potential illness.

Self-compassion theory suggests that when individuals are experiencing pain or difficulty, they adopt a supportive and non-judgmental attitude towards themselves, thereby playing a protective role for their physical and mental health (Neff, 2023). From the perspective of protection motivation theory, self-compassion can be viewed as a key component of coping appraisal: by reducing harsh self-criticism and fostering a kinder, more balanced self-view, it strengthens self-efficacy (confidence in one’s ability to cope) and response efficacy (belief that adaptive behaviors such as exercise will be beneficial) (Chishima et al., 2018).

Furthermore, protection motivation theory posits that the greater the perceived threat, the more likely individuals are to develop an awareness of protective behavior, and this awareness in turn activates, sustains, and guides coping responses (Milne et al., 2000; Floyd et al., 2000). However, excessive threat may lead individuals to adopt a range of ineffective coping strategies such as avoidance or denial, rather than engaging in positive protective behaviors (Witte, 1992). In our model, coping strategies represent the concrete coping responses selected to deal with health-related threat, and physical activity is treated as an adaptive protective behavior that individuals may choose to implement as a way of managing perceived risk and anxiety. Self-compassion can provide important support in this process: by increasing self-compassion, individuals may be better able to assess threats with calmness and acceptance, reduce the interference of emotional reactions with rational decision-making, and thus be more likely to adopt health recommendations derived from protection motivation theory (Vos et al., 2021). Therefore, this study argues that when young and middle-aged employees experience maladjustment in their physical or mental health or have elevated levels of health anxiety, higher self-compassion may help them evaluate threats more calmly, develop a stronger and healthier sense of protection, and consequently adopt appropriate coping strategies (such as participating in physical activity) to prevent health anxiety or reduce its negative impact.

### 2.2 Concepts

#### 2.2.1 Health anxiety

Health anxiety refers to excessive concern about illness, typically manifesting in two forms: hypochondriasis—the mistaken belief of already having a serious illness—and disease phobia—the fear of developing one. Both arise from the misinterpretation of bodily sensations (Sunderland et al., 2013). As a recognized psychological disorder, health anxiety persists even when medical evaluations return normal results (Lebel et al., 2020). Research has shown that 23.4% of frontline workers report symptoms of anxiety, with 7.5% experiencing moderate to severe levels of anxiety (Duplaga and Grysztar, 2021). In this study, health anxiety is specifically defined in the context of young and middle-aged employees. Employees with health anxiety often experience psychological symptoms such as depression, anxiety, fear, social withdrawal, and obsessive-compulsive tendencies (Heinen et al., 2021). Physiological manifestations may involve sleep disturbances, fatigue, and dizziness. While these symptoms are not typically life-threatening, they can substantially diminish quality of life and negatively impact both the individuals and their families (Wang et al., 2023). Moreover, fear and anxiety about potential illness may impair employees’ problem-solving abilities and reduce work efficiency (Korkmaz et al., 2020).

#### 2.2.2 Self-compassion

Although rooted in Buddhism, self-compassion has been adapted as a secular psychological construct, defined as responding to personal setbacks with understanding rather than self-criticism (Neff, 2011). It comprises three elements: self-kindness, recognition of shared human experience, and emotional balance (Barnard and Curry, 2011). Research links self-compassion to reduced depression and anxiety and increased life satisfaction (MacBeth and Gumley, 2012). In workplace contexts, it is associated with lower emotional exhaustion, burnout, and greater wellbeing (Kotera and Van Gordon, 2021). Recent studies further suggest that self-compassion fosters emotional resilience and motivation, enhancing job performance and buffering stress from high job demands (Jennings et al., 2023; Moreno-Jiménez et al., 2023).

#### 2.2.3 Coping strategies

Coping refers to the cognitive and behavioral strategies individuals use to manage stressors perceived as overwhelming (Donnellan et al., 2006). These strategies are essential for maintaining psychological and physical wellbeing when facing challenges, threats, or stressors (Ho, 2019; Wechsler, 1995). This study focuses on young and middle-aged employees, thus coping strategies are examined from a work-related perspective. In occupational settings, coping strategies involve techniques used to balance work and life through cognition, behavior, and interpersonal relationships (Zheng et al., 2016). Ben-Ezra and Hamama-Raz (2021) suggest that employees often bear additional pressures from their roles within the family, and may cope by avoiding stressors, distancing themselves, seeking emotional support, venting, or finding joy in specific circumstances. Other research has shown that when facing work stress and burnout, employees also adopt positive coping strategies. For instance, social workers under resource constraints may seek organizational support, improve professional skills, or build relationships, while healthcare professionals tend to rely on self-compassion and teamwork to manage stress and burnout (Zhang, 2022; Vagni et al., 2022).

#### 2.2.4 Physical activity

Physical activity, defined as planned, structured, and repetitive movement aimed at improving or maintaining fitness (Caspersen et al., 1985), offers not only physical benefits but also enhances self-esteem, fosters social interaction, reduces stress, and promotes overall mental and physical health (Chen and Sun, 2012). Anaza and and McDowell (2013) suggest that physical activity enhances life satisfaction by promoting well-being, mental clarity, and social connection. In the workplace, poor health can hinder performance, whereas physical activity is linked to better health-related quality of life and productivity (Pereira et al., 2015). Interventions like workplace yoga have also been shown to support physical, emotional, and mental well-being (Garcia et al., 2021). Moreover, it offers a practical countermeasure to the negative health impacts of prolonged sedentary work (Michalchuk et al., 2022).

### 2.3 Hypotheses

#### 2.3.1 Health anxiety, self-compassion, coping strategies

Research by Jungmann and Witthöft (2020) indicates that heightened health-related worries can lead to psychological distress, including stress, avoidance behaviors, and persistent negative thinking patterns such as chronic pessimism. These outcomes are often linked to maladaptive or even harmful self-protective behaviors. Wen et al. (2021) found that health anxiety may increase the risk of cardiovascular disease and indirectly raise mortality. In contrast, self-compassion serves as a protective factor, reducing symptoms of depression and anxiety (Pauley and McPherson, 2010). Van Dam et al. (2011) describe self-compassion as a constructive psychological resource that helps individuals replace self-criticism and rigid self-control with greater emotional flexibility and self-acceptance.

Dundas et al. (2017) found that self-compassion enhances adaptive self-regulation, including greater self-efficacy and impulse control, while reducing self-criticism and negative rumination. Similarly, self-compassion interventions have been shown to support mental well-being and promote healthier choices under stress, especially among individuals managing demanding health behaviors (Biber and Ellis, 2017). Therefore, research situated in workplace settings suggests that under high-pressure environments, employees are becoming increasingly aware of their psychological well-being and actively seek stress management strategies—among which self-compassion is notable (Meng et al., 2020; Lathren et al., 2024). Research also suggests that heightened health anxiety can increase employees’ awareness of their physical and mental health. Under stress, for example, healthcare workers may adopt self-compassion practices like meditation or yoga to manage anxiety and enhance well-being (Taylor et al., 2022; Wagner et al., 2024).

According to protection motivation theory, heightened perception of health threats increases fear, which in turn motivates protective behaviors (Milne et al., 2000). When individuals recognize the risks of cardiovascular disease and their own vulnerability, they are more likely to adopt coping strategies to protect their health (Bui et al., 2013). Subsequent studies have shown that low levels of coping awareness and emotional regulation are among the contributing factors to heightened health anxiety. Therefore, individuals with health anxiety often adopt coping strategies to counter its negative effects (Barron Millar et al., 2021; Vintila et al., 2023). Such strategies are essential for reducing stress and negative emotions, promoting healthier behaviors, and preventing harmful outcomes like physical inactivity (Pasha et al., 2022).

Studies show that individuals with higher self-compassion are more likely to face adversity with kindness and adopt proactive coping strategies in response to potential threats (Allen and Leary, 2010). Self-compassion also enhances coping effectiveness by promoting adaptive responses and reducing reliance on maladaptive strategies, thereby buffering the impact of stress (Lloyd et al., 2019). In contrast, individuals with lower self-compassion are more likely to adopt ineffective coping behaviors and show less motivation for self-growth under stress (Chwyl et al., 2020). As a positive psychological resource, self-compassion fosters constructive coping strategies (Wang Y. et al., 2024). Based on this literature, the following hypotheses are proposed:

*Hypothesis 1 (H1)*: Health anxiety is positively associated with self-compassion.

*Hypothesis 2 (H2)*: Health anxiety is positively associated with coping strategies.

*Hypothesis 3 (H3)*: Self-compassion is positively associated with coping strategies.

#### 2.3.2 Self-compassion, coping strategies, physical activity

Emerging research indicates that self-compassion supports physical health by promoting positive lifestyle behaviors, including regular exercise, balanced nutrition, and adequate sleep (Dunne et al., 2016). Individuals with higher levels of self-compassion are more likely to adopt and sustain health-enhancing habits, benefiting both mental and physical well-being (Ferrari et al., 2017). They also demonstrate greater autonomy in managing their health, making them more inclined to engage in consistent physical activity (Huang et al., 2021). Additionally, self-compassion has been associated with intrinsic motivation and a proactive attitude—both critical for maintaining long-term engagement in physical activity (Pastore et al., 2023). It further facilitates the removal of psychological barriers and supports adaptive behavioral responses, thereby encouraging the initiation and continuation of physical activity (Zhang et al., 2025).

Wandel and Roos (2005) found that physical activity improves both mental and physical health among professionals. As a result, more employees are using exercise to cope with work-related stress and unhealthy routines. Under pressure, physical activity offers a healthier alternative to maladaptive responses and has been shown to alleviate conditions such as obesity, cardiovascular disease, and hypertension—making it an effective and sustainable coping approach (Cairney et al., 2014). Dahlstrand et al. (2021) further noted that the more time individuals devote to physical activity, the greater the reduction in stress and physical symptoms. Moreover, physical activity acts as a protective factor, enhancing life satisfaction and reducing perceived stress (Shpakou et al., 2022). Based on this evidence, the following hypotheses are proposed:

*Hypothesis 4 (H4)*: Self-compassion is positively associated with physical activity.

*Hypothesis 5 (H5)*: Coping strategies are positively associated with physical activity.

#### 2.3.3 The mediating effect

Higher self-compassion is linked to lower levels of loneliness, sadness, and other negative emotions. Self-compassion also helps manage health anxiety and promotes healthy behaviors like physical activity, thereby reducing disease risk. When individuals perceive a threat to their health, they may increase their level of self-compassion to buffer against anxiety and make healthier behavioral choices (Phillips and Hine, 2021; Terry et al., 2013). Excessive self-criticism and self-blame are major contributors to anxiety, depression, and broader mental health issues (Hughes et al., 2021). Self-compassion, by encouraging self-acceptance, can counter these tendencies and reduce the likelihood of mental illness, thereby easing the distress associated with anxiety-related conditions. Studies have shown that health anxiety triggers early warning responses to potential threats, motivating individuals to adopt coping strategies that protect them—often through healthier behaviors (Asmundson and Taylor, 2020). Garbóczy et al. (2021) further demonstrated that in high-stress environments, increased health anxiety leads to greater frequency of coping strategy use.

Meanwhile, self-compassion supports emotional regulation during setbacks in physical activity, enhancing motivation and perseverance. Enhanced self-regulation strengthens self-efficacy—one’s confidence in managing tasks under specific conditions—which in turn supports sustained engagement in physical activity. Self-compassion supports both the initiation and long-term maintenance of physical activity (Zhang et al., 2023; Zhang et al., 2025). In turn, exercise boosts endorphins and brain-derived neurotrophic factors, enhancing mood and reducing stress. Physical activity also has fewer side effects and can enhance health outcomes while reducing reliance on medication, making it a preferred health and social coping strategy for many employees (Wandel and Roos, 2005; Faulkner et al., 2020; Ai et al., 2021). Based on this evidence, the following mediation hypothesis is proposed:

*Hypothesis 6 (H6)*: Self-compassion and coping strategies mediate the relationship between health anxiety and physical activity.

**Figure 1:**
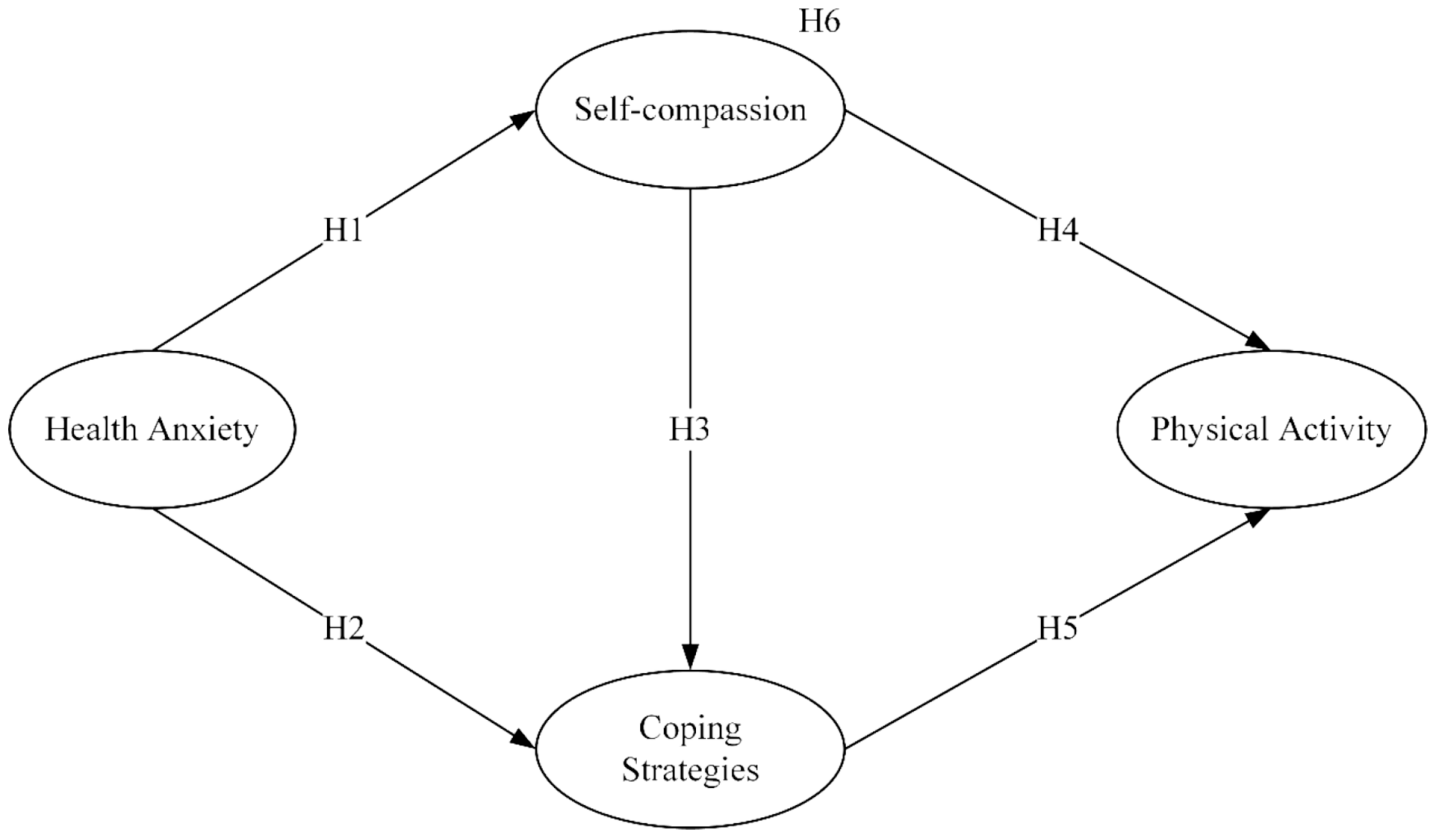
Hypothetical model.

A summary of all hypotheses is illustrated in Figure 1.

## 3 Methodology

### 3.1 Participants and procedures

This study used snowball and convenience sampling to administer a questionnaire survey among young (18–34) and middle-aged (35–44) employees from diverse industry sectors. Drawing on the age group definitions used by Mullet et al. (1998) and Monod et al. (2021), individuals aged 18–34 were classified as young adults, while those aged 35–44 were classified as middle-aged. These groups were designated as the target population of the study.

To collect the data, we conducted a survey between January and February 2025 among employees working in the public service, manufacturing, service, and information technology sectors in Shandong, Jiangxi, and Anhui provinces in China. Potential participants were approached via social media platforms, including WeChat, Tencent QQ, and Weibo, and were first asked whether they were willing to take part in the study. Those who agreed received a link to the online questionnaire. Before starting the survey, all participants were informed of the research purpose, assured that participation was entirely voluntary, and told that their responses would be kept confidential and used only for research. As an incentive, respondents who completed the questionnaire were offered either a small shopping voucher or a gym pass. Participants were also encouraged to forward the questionnaire link to their colleagues and friends. In total, 456 questionnaires were returned; after excluding cases with unusually short completion times or identical responses across items, 423 valid questionnaires remained, yielding a valid response rate of 93.8%.

### 3.2 Instruments

The questionnaire consisted of five sections. The first gathered demographic data, including age, gender, occupation, and industry. The second section uses five items from the scale developed by Bot et al. (2014) to assess health anxiety among young and middle-aged workers (e.g., “I notice aches and pain less/as much/more than other people”). The third section adopts six items from the revised scale by Neff et al. (2021) to collect data on self-compassion (e.g., “I’m giving myself the caring and tenderness I need”). The fourth section includes eight items across two dimensions from the scale developed by Tobin et al. (1989), aiming to evaluate coping strategies among young and middle-aged employees (e.g., “I try to let my emotions out”). The fifth section uses three items from the revised scale by Liang (1994) to assess the level of physical activity (e.g., “How often do you do physical activity every month/week?”).

When using Western-developed scales, we either adopted existing validated Chinese versions or, where such versions were not available, followed a standard translation and back-translation procedure to ensure linguistic and conceptual equivalence. These Chinese versions of the instruments have been applied in studies conducted in the Chinese context by scholars such as Wang Z. et al. (2024), Yu et al. (2025), and Wang et al. (2022), and have demonstrated satisfactory reliability and validity. Except for demographic items, all questions were rated on a five-point Likert scale. Physical activity intensity, for instance, was scored from 1 (light activity) to 5 (vigorous activity of long duration), allowing for quantitative analysis.

### 3.3 Data analysis

After data cleaning and quality checks, 423 valid responses were retained for analysis. Descriptive statistics and assumption testing were performed using SPSS 25. To test the research model, structural equation modeling (SEM) was conducted with SmartPLS 4.1.0, applying the partial least squares (PLS) method, which is well-suited for complex models with multiple constructs. Model evaluation focused on convergent and discriminant validity, assessed through factor loadings and average variance extracted (AVE). SEM was used to examine the interrelationships among key variables in the conceptual framework.

## 4 Results

### 4.1 Sample characteristics

Table 1 presents the following demographic characteristics: the proportion of male and female participants was roughly balanced; among the participants, those with a monthly income of 5,001–10,000 CNY accounted for a larger proportion than other income groups, reaching 45.6% of the total sample. Scholars have suggested that this pattern mainly arises because people in this income bracket are generally able to meet their basic living needs and therefore have more resources to devote to health-related concerns (Koivusilta et al., 2024). In addition, 69.3% of participants rated their own health status as good. Scholars argue that this may be because the survey sample mainly comprised young adults aged 18–34 years, who accounted for 73.8% of the respondents. A study focusing on young people has further shown that this group tends to have physiological advantages such as stronger cellular regeneration capacity, a healthier immune system, and a relatively lower incidence of chronic diseases. These advantages enable young people to show greater resilience when facing everyday health challenges and thus make them more inclined to evaluate their own health status positively (Howie et al., 2020).

**Table 1:** Demographic characteristics.

Category	Item	*n*	%
Gender	Male	215	50.8
	Female	208	49.2
Age	18–34	312	73.8
	35–44	111	26.2
Education	High school or below	73	17.3
	Associate degree	116	27.4
	Bachelor’s degree	183	43.2
	Master’s degree or above	51	12.1
Industry	Public services	167	39.5
	Manufacturing	56	13.2
	Service industry	43	10.2
	Information technology	74	17.5
	Other	83	19.6
Monthly income	≤ 5,000 CNY	167	39.5
	5,001–10,000 CNY	193	45.6
	10,001–15,000 CNY	28	6.6
	≥ 15,001 CNY	35	8.3
Self-reported health status	Good	293	69.3
	Average	121	28.6
	Poor	9	2.1

As shown in Table 2, participants reported moderate levels of health anxiety, self-compassion, coping strategies, and physical activity. Skewness and kurtosis values (ranging from −1.41 to 0.20) indicated no severe deviations from univariate normality. Pearson correlations showed that health anxiety was positively associated with self-compassion, coping strategies, and physical activity (rs = 0.46–0.69, ps < 0.01), providing initial support for the hypothesized relationships.

**Table 2:** Descriptive statistics and Pearson correlations.

Variable	M	SD	Skewness	Kurtosis	1	2	3	4
1. Health anxiety	2.86	0.96	0.01	−0.84	–			
2. Self-compassion	3.01	0.90	0.09	−0.16	0.62**	–		
3. Coping strategies	3.15	1.06	−0.16	−0.67	0.69**	0.81**	–	
4. Physical activity	2.71	1.23	0.20	−1.41	0.58**	0.46**	0.50**	–

M, mean; SD, standard deviation. Values on the diagonal are omitted. All coefficients are Pearson correlations. **p < 0.01 (two-tailed).

### 4.2 Assessment of the measurement model reliability and validity

In the initial stage of PLS-SEM analysis, internal reliability was assessed using Cronbach’s alpha, composite reliability (CR), and rho-A. All constructs showed strong reliability, with the lowest Cronbach’s alpha at 0.894 and CR at 0.922. Convergent validity, evaluated via average variance extracted (AVE), exceeded the recommended 0.50 threshold for all constructs, confirming adequacy. Detailed results are presented in Table 3.

**Table 3:** Reliability and validity test.

Items	Loading	Cα	CR	AVE
Health anxiety (HA)		0.894	0.922	0.704
HA1	0.870			
HA2	0.851			
HA3	0.871			
HA4	0.754			
HA5	0.843			
Coping strategies (CS)		0.969	0.974	0.821
Problem-focused engagement (PFE)		0.947	0.962	0.862
PFE1	0.911			
PFE2	0.919			
PFE3	0.944			
PFE4	0.939			
Problem-focused disengagement (PFD)		0.955	0.968	0.882
PFD1	0.915			
PFD2	0.955			
PFD3	0.943			
PFD4	0.943			
Self-compassion (SC)		0.980	0.984	0.911
SC1	0.944			
SC2	0.953			
SC3	0.961			
SC4	0.957			
SC5	0.953			
SC6	0.957			
Physical activity (PA)		0.898	0.937	0.831
PA1	0.925			
PA2	0.913			
PA3	0.897			

Cα, Cronbach’s alpha; AVE, average variance extracted; CR, composite reliability.

Discriminant validity was evaluated using the Heterotrait–Monotrait Ratio (HTMT). According to the criteria by Clark and Watson (2016) and Lee et al. (2011), HTMT values below 0.85 indicate acceptable discriminant validity. As shown in Table 4, all values met this criterion, confirming satisfactory discriminant validity across constructs.

**Table 4:** Discriminant validity test.

Variable	CS	HA	PA	SC
CS				
HA	0.737			
PA	0.534	0.645		
SC	0.833	0.663	0.487	

HA, health anxiety; CS, coping strategies; SC; PA, physical activity.

### 4.3 Hypothesis testing results

In line with our theoretical model, we specified a fully mediated structural model without a direct path from health anxiety to physical activity; therefore, the direct effect was fixed to zero and not estimated.

**Figure 2:**
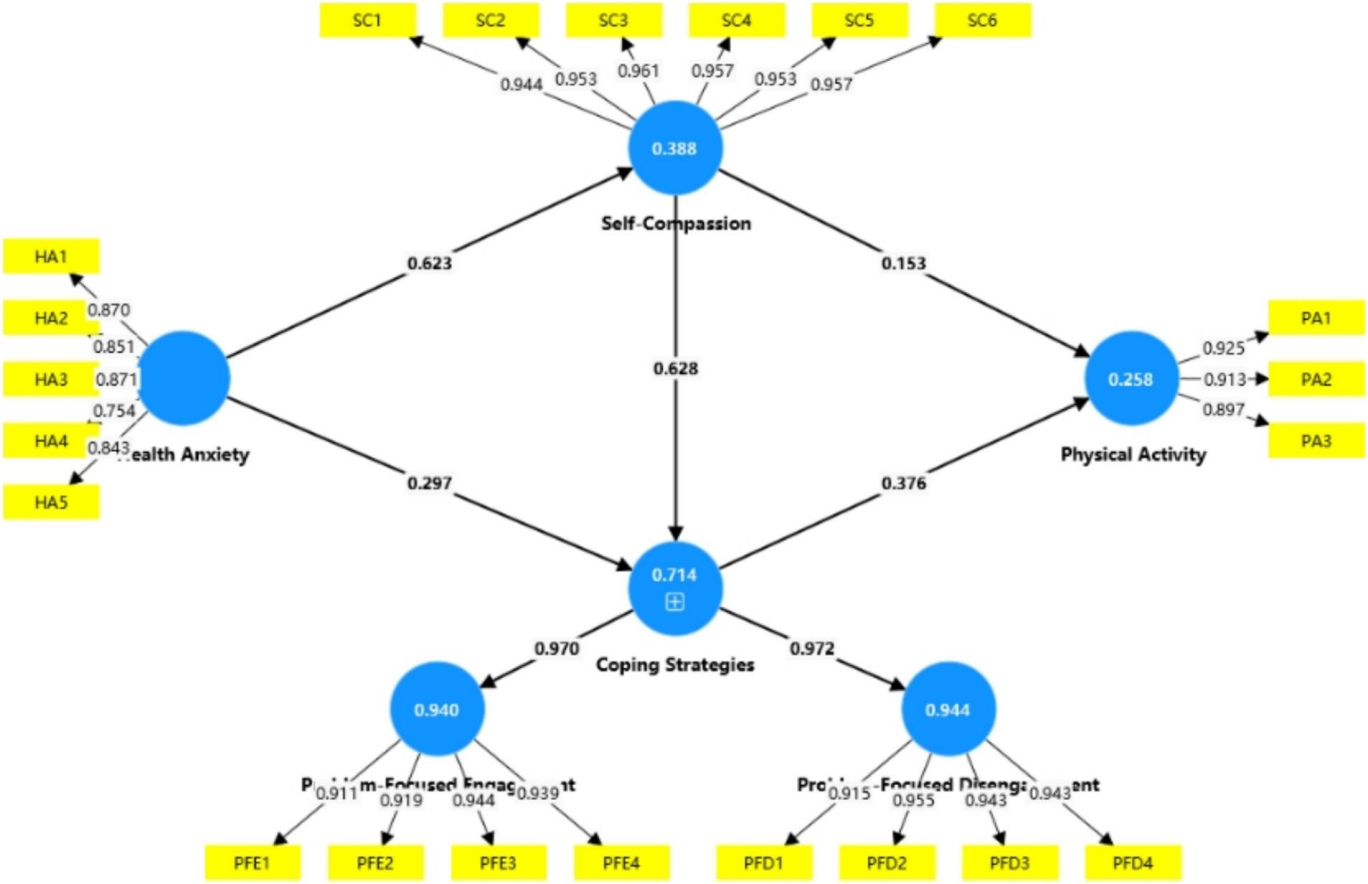
Structural path model.

Model fit was assessed using the Standardized Root Mean Square Residual (SRMR), with values below 0.08 indicating acceptable fit. The SRMR for this model was 0.052, suggesting a satisfactory fit. Structural path results are summarized in Table 5 and illustrated in Figure 2. Health anxiety was positively associated with self-compassion (β = 0.623, t = 19.026, *p* < 0.001), supporting H1, and with coping strategies (β = 0.297, t = 5.534, *p* < 0.001), supporting H2. Self-compassion was significantly related to coping strategies (β = 0.628, t = 11.061, *p* < 0.001), confirming H3. Additionally, self-compassion (β = 0.153, t = 2.531, *p* < 0.05) and coping strategies (β = 0.376, t = 5.796, *p* < 0.001) both positively predicted physical activity, supporting H4 and H5, respectively.

**Table 5:** Path coefficients.

No	Path	β	SEs	T statistics	*p* values	LLIC	ULIC
H1	HA → SC	0.623	0.033	19.026	0.000	0.557	0.685
H2	HA → CS	0.297	0.054	5.534	0.000	0.196	0.405
H3	SC → CS	0.628	0.057	11.061	0.000	0.513	0.732
H4	SC → PA	0.153	0.061	2.531	0.011	0.040	0.281
H5	CS → PA	0.376	0.065	5.796	0.000	0.239	0.497

HA, health anxiety; CS, coping strategies; SC, self-compassion; PA, physical activity.

### 4.4 Mediation analysis

To test the mediating roles of self-compassion and coping strategies between health anxiety and physical activity, a bootstrapping procedure with 5,000 resamples and 95% confidence intervals was used, following Bollen and Stine (1990). As shown in Table 6, health anxiety had a significant indirect effect on physical activity via self-compassion (HA → SC → PA) and via coping strategies (HA → CS → PA), as the 95% bootstrap confidence intervals for both paths did not include zero. In addition, the serial indirect path through both mediators (HA → SC → CS → PA) was also significant. Taken together, the total indirect effect of health anxiety on physical activity was significant (β = 0.354, *p* < 0.001), providing support for the fully mediated model.

**Table 6:** Mediation analysis.

No	Path	Effect	Boot SE	T statistics	*P* values	Boot LLIC	Boot ULCI
H6	HA → SC → PA	0.096	0.040	2.414	0.016	0.022	0.177
	HA → CS → PA	0.111	0.033	3.416	0.001	0.055	0.183
	HA → SC → CS → PA	0.147	0.026	5.650	0.000	0.103	0.206
	Total indirect effect of HA → PA	0.354	0.033	10.675	0.000	0.285	0.415

HA, health anxiety; CS, coping strategies; SC, self-compassion; PA, physical activity.

## 5 Discussion

### 5.1 Theoretical contributions

Grounded in protection motivation theory and informed by key concepts from self-compassion theory, this study examines the association between health anxiety and physical activity among young and middle-aged employees, thereby extending these theoretical perspectives to the context of occupational health. While prior research has largely focused on how physical activity helps alleviate health anxiety, most studies have not explored this relationship specifically within working populations. For instance, Anderson and Shivakumar (2013), McDowell et al. (2019), and Wang et al. (2023) found a negative association between physical activity and health anxiety. Likewise, Kirmizi et al. (2021) and Carter et al. (2021) noted that physical activity reduces musculoskeletal symptoms and enhances self-efficacy, both of which may help alleviate anxiety. However, little attention has been given to how health anxiety itself might influence physical activity engagement—particularly among younger and less experienced employees, who are more prone to anxiety (Mokhtari et al., 2020; Duplaga and Grysztar, 2021). Moreover, despite growing evidence of their importance, few studies have explored the mediating roles of self-compassion and coping strategies in the relationship between health anxiety and physical activity (Barron Millar et al., 2021; Vintila et al., 2023).

This study examined the relationship between health anxiety and physical activity among young and middle-aged employees, revealing several significant pathways. First, health anxiety was positively associated with self-compassion (see Figure 2), aligning with findings by Meng et al. (2020), Taylor et al. (2022), Lathren et al. (2024) and Wagner et al. (2024). This suggests that individuals experiencing health anxiety may become more attuned to their well-being and adopt more self-compassionate attitudes. Similarly, a positive association was found between health anxiety and coping strategies, supporting the work of Bui et al. (2013), Barron Millar et al. (2021), and Vintila et al. (2023) indicating that employees may respond to health-related stress through active and constructive coping behaviors.

The model also showed that self-compassion was positively associated with physical activity engagement, consistent with findings by Dunne et al. (2016), Rebar et al. (2016), Ferrari et al. (2017), and Huang et al. (2021) who noted that self-compassionate individuals are more likely to sustain regular exercise for overall well-being. Coping strategies were likewise positively associated with physical activity, aligning with Wandel and Roos (2005) and Cairney et al. (2014). These results suggest that employees may often use physical activity as a coping mechanism to counter work-related stress and sedentary routines, while reducing reliance on maladaptive behaviors linked to increased health risks, such as obesity or cardiovascular disease (Firth et al., 2020). In addition, this study found that the path coefficient between coping strategies and physical activity (β = 0.376) was higher than that between self-compassion and physical activity (β = 0.153). One plausible explanation is that coping strategies function as more proximal, behavior-focused regulators of physical activity: they directly guide how individuals respond to stress in everyday life and can help them anticipate and overcome practical barriers (e.g., lack of time, fatigue), thereby increasing their level of exercise participation (Braun et al., 2024; Braun et al., 2023). By contrast, self-compassion represents a higher-order psychological resource that may influence physical activity mainly through indirect pathways—for instance, by fostering adaptive coping patterns and enhancing self-efficacy rather than prompting immediate behavioral change. For example, Zhang et al. (2023) and Zhang et al. (2025) found that self-compassion can enhance individuals’ self-efficacy, which in turn promotes greater involvement in physical activity.

Notably, while previous studies often examined self-compassion and coping strategies as direct predictors of physical activity (Zhang et al., 2023; Phillips and Hine, 2021; Murphy et al., 2012; Pasha et al., 2022), the current study highlights their mediating roles. The findings indicate that self-compassion and coping strategies mediate the relationship between health anxiety and physical activity, highlighting correlational pathways and underscoring the importance of psychological mechanisms in shaping adaptive health behaviors in the workplace.

### 5.2 Practical implications

In light of these correlational findings, young and middle-aged employees experiencing health anxiety should be encouraged to adopt healthy coping strategies in place of maladaptive ones that may harm their physical wellbeing. Physical activity is a healthy coping strategy that supports both mental and physical health while helping to reduce the risk of obesity and cardiovascular disease. Additionally, it provides a supportive social environment that can enhance interpersonal communication skills. To encourage more individuals with health anxiety to adopt physical activity as a coping mechanism, this study offers recommendations across three levels: government, organizational, and individual.

At the government level, it is recommended that public sports facilities—such as fitness centers, basketball courts, and badminton courts—be built in office-dense areas to lower the threshold for employees to engage in exercise. In practical terms, local governments can incorporate workplace fitness infrastructure into urban planning guidelines and allocate earmarked budgets for constructing or renovating such venues near major business districts. Government-managed sports venues should be opened free of charge during specific time slots on working days (e.g., lunch breaks and weekday evenings). Clear time slots can be announced in advance and coordinated with employers to allow staff to use these facilities during flexible breaks. Authorities can also launch initiatives such as “National Fitness Campaigns” and “Workplace Health Challenges” to promote participation. For example, governments can organize city-wide step-count competitions or “active commuting” weeks, provide standardized toolkits for enterprises (posters, online platforms, registration forms), and publish rankings or awards to increase visibility. Finally, governments should regularly recognize and commend enterprises that have made outstanding contributions to employees’ health, thereby creating a demonstration effect. This can be implemented through annual “Healthy Workplace” certifications or awards linked to modest financial or tax incentives, encouraging more organizations to invest in employee physical activity.

At the organizational level, employers are advised to set up fitness rooms, yoga studios, or similar facilities within office spaces, or to partner with nearby gyms to provide employees with free or discounted memberships. Human resource departments can include exercise programs in formal wellness policies, specifying available facilities, subsidies, and eligibility criteria. Organizations can encourage and financially support employees to establish running, yoga, basketball, badminton and other sports clubs to promote exercise-based social interaction among colleagues. In practice, companies can provide small annual budgets for each club (e.g., for equipment or event fees) and designate a coordinator responsible for scheduling regular sessions. Regular company sports meets and team-building activities centered on physical activity should be organized, with rewards for employees who demonstrate sustained participation and improvement. These activities can be scheduled quarterly, integrated into performance or recognition systems (e.g., “active employee” awards), and monitored through simple sign-up or attendance records.

At the individual level, employees should set specific and achievable exercise goals and maintain them over the long term. For example, they may start with 10–15 min of brisk walking three times per week and gradually increase duration or intensity. They are encouraged to try different types of physical activity to identify forms of exercise that genuinely interest them. Engaging in physical activity together with colleagues, friends, or family members can provide mutual supervision and encouragement. Employees can make fixed “exercise appointments” with others (e.g., after work on two set weekdays) to enhance adherence. Individuals can also use fitness trackers and exercise apps to record their progress, take part in online challenges, and learn scientifically informed exercise methods. When possible, they are advised to seek guidance from qualified fitness professionals or evidence-based health resources to avoid injury and to match exercise intensity to their health status.

In conclusion, promoting physical activity as a coping strategy for young and middle-aged employees experiencing health anxiety may enhance their mental and physical well-being and may also support organizational sustainability. Accordingly, both governments and employers should acknowledge the value of physical activity and take active measures to create supportive environments that facilitate employee participation in regular exercise.

### 5.3 Limitations

First, this study adopted a cross-sectional survey design, which can reveal associations among health anxiety, self-compassion, coping strategies, and physical activity but does not allow causal inferences to be drawn. Future research could therefore use multi-wave longitudinal designs (e.g., assessing health anxiety and self-compassion at Time 1, coping strategies at Time 2, and physical activity at Time 3) and include moderating variables such as health literacy or social support to clarify temporal ordering. In addition, this study relied on self-report data from young and middle-aged employees, which may be subject to recall bias and under- or over-reporting of physical activity. Physical activity was also assessed using a brief self-report scale without objective indicators (e.g., accelerometers), which may oversimplify the construct. Future studies should consider combining self-report with wearable activity monitors and, rather than treating health anxiety as a unidimensional construct, examine how different dimensions (cognitive, behavioral, emotional, perceptual) are associated with physical activity.

Second, the data in this study were collected using snowball and convenience sampling, with participants recruited primarily via social media platforms, and mainly from employees in three eastern Chinese provinces (Shandong, Anhui, and Jiangxi). Although these methods reduced the complexity of data collection, the reliance on online recruitment and the uneven distribution across industries (e.g., a higher proportion of public-sector employees and a relatively small proportion from the service sector) may have biased the sample toward individuals with higher health awareness and limited the external validity of the findings. Moreover, because all three provinces are located in eastern China and share relatively similar patterns of economic development and access to health resources, the sample may not fully capture the diversity of coping styles and self-compassion levels that might be observed in other regions (e.g., western or rural areas). Consequently, future research should further refine the sampling strategy, broaden the geographical coverage, and include a wider range of occupational groups, so as to improve the representativeness and generalizability of the results.

Third, China is a large developing country with multiple coexisting cultural traditions, and there are substantial regional differences in both economic development and local culture. These cultural and regional differences may influence how individuals interpret health-related threats, which coping strategies they consider acceptable or effective, and the extent to which they view self-compassion as a legitimate way of dealing with stress. The interplay of these contextual factors may affect the consistency of measurements for variables such as coping strategies and self-compassion, as these factors are complex and not independent of one another. Future studies should therefore seek to clarify the influence of these contextual characteristics in order to better understand how they shape individuals’ coping strategies and self-compassion.

Finally, this study mainly focused on the positive pathways through which self-compassion and coping strategies promote physical activity. Although this approach provides valuable insights, it did not consider the role of potential covariates (such as health literacy) in the relationship between health anxiety and physical activity among young and middle-aged employees. Future research could incorporate such variables as moderators to further explore their effects. In addition, this study did not examine possible reverse or reciprocal relationships. Subsequent studies could therefore investigate the bidirectional or cyclical associations among health anxiety, self-compassion, coping strategies, and physical activity from alternative perspectives, thereby offering a more comprehensive understanding of these four variables.

## 6 Conclusion

The findings align with the study’s objectives, demonstrating that physical activity can serve as an effective coping strategy for alleviating health anxiety among young and middle-aged employees. The results further indicate that individuals with higher levels of self-compassion and adaptive coping are more likely to engage in physical activity in response to health-related stress. Based on these insights, the study recommends promoting physical activity as a constructive response to health anxiety, encouraging long-term exercise habits to support both psychological and physical well-being. Furthermore, it calls on governments and employers to invest in sports infrastructure and foster an organizational culture that supports physical activity. Expanding access to exercise opportunities not only improves employee health and resilience but also enhances workplace performance by broadening the range of effective coping resources.

## Data Availability

The raw data supporting the conclusions of this article will be made available by the authors, without undue reservation.

## References

[ref1] Ai X. Yang J. Lin Z. Wan X. (2021). Mental health and the role of physical activity during the Covid-19 pandemic. Front. Psychol. 12:759987. doi: 10.3389/fpsyg.2021.759987, PMID: 34744938 PMC8565623

[ref2] Allen A. B. Leary M. R. (2010). Self-compassion, stress, and coping. Soc. Personal. Psychol. Compass 4, 107–118. doi: 10.1111/j.1751-9004.2009.00246.x, PMID: 20686629 PMC2914331

[ref3] Anaza E. And Mcdowell J. (2013). An investigation of constraints restricting urban Nigerian women from participating in recreational sport activities. J. Leis. Res. 45, 324–344. doi: 10.18666/jlr-2013-v45-i3-3154

[ref4] Anderson E. H. Shivakumar G. (2013). Effects of exercise and physical activity on anxiety. Front. Psych. 4:27. doi: 10.3389/fpsyt.2013.00027, PMID: 23630504 PMC3632802

[ref5] Asmundson G. J. G. Taylor S. (2020). How health anxiety influences responses to viral outbreaks like Covid-19: what all decision-makers, health authorities, and health care professionals need to know. J. Anxiety Disord. 71:102211. doi: 10.1016/j.janxdis.2020.102211, PMID: 32179380 PMC7271220

[ref6] Barnard L. K. Curry J. F. (2011). Self-compassion: conceptualizations, correlates, & interventions. Rev. Gen. Psychol. 15, 289–303. doi: 10.1037/a0025754

[ref7] Barron Millar E. Singhal D. Vijayaraghavan P. Seshadri S. Smith E. Dixon P. . (2021). Health anxiety, coping mechanisms and Covid 19: an Indian community sample at week 1 of lockdown. PLoS One 16:e0250336. doi: 10.1371/journal.pone.0250336, PMID: 33882109 PMC8059846

[ref8] Ben-Ezra M. Hamama-Raz Y. (2021). Social workers during Covid-19: do coping strategies differentially mediate the relationship between job demand and psychological distress? Br. J. Soc. Work. 51, 1551–1567. doi: 10.1093/bjsw/bcaa210

[ref9] Biber D. D. Ellis R. (2017). The effect of self-compassion on the self-regulation of health behaviors: a systematic review. J. Health Psychol. 24, 2060–2071. doi: 10.1177/1359105317713361, PMID: 28810473

[ref10] Bollen K. A. Stine R. (1990). Direct and indirect effects: classical and bootstrap estimates of variability. Sociol. Methodol. 20, 115–140. doi: 10.2307/271084

[ref11] Bot A. G. J. Becker S. J. E. Bruijnzeel H. Mulders M. A. M. Ring D. Vranceanu A.-M. (2014). Creation of the abbreviated measures of the pain catastrophizing scale and the short health anxiety inventory: the Pcs-4 and Shai-5. J. Musculoskelet. Pain. 22, 145–151. doi: 10.3109/10582452.2014.88302

[ref12] Braun M. Crombez G. De Backere F. Tack E. De Paepe A. L. (2024). An analysis of physical activity coping plans: mapping barriers and coping strategies based on user ratings. Health Psychol. Behav. Med. 12:2434140. doi: 10.1080/21642850.2024.2434140, PMID: 39628965 PMC11613413

[ref13] Braun M. Schroé H. De Paepe A. L. Crombez G. (2023). Building on existing classifications of behavior change techniques to classify planned coping strategies: physical activity diary study. Jmir Form. Res. 7:e50573. doi: 10.2196/50573, PMID: 38109171 PMC10758936

[ref14] Bui L. Barbara M. And Mccaffery K. (2013). Protection motivation theory and physical activity in the general population: a systematic literature review. Psychol. Health Med. 18, 522–542. doi: 10.1080/13548506.2012.749354 23324044

[ref15] Cairney J. Kwan M. Y. Veldhuizen S. Faulkner G. E. (2014). Who uses exercise as a coping strategy for stress? Results from a national survey of Canadians. J. Phys. Act. Health 11, 908–916. doi: 10.1123/jpah.2012-0107, PMID: 23493043

[ref16] Carter T. Pascoe M. Bastounis A. Morres I. D. Callaghan P. Parker A. G. (2021). The effect of physical activity on anxiety in children and young people: a systematic review and meta-analysis. J. Affect. Disord. 285, 10–21. doi: 10.1016/j.jad.2021.02.026, PMID: 33618056

[ref17] Caspersen C. J. Powell K. E. Christenson G. M. (1985). Physical activity, exercise, and physical fitness: definitions and distinctions for health-related research. Public Health Rep. 100, 126–131. 3920711 PMC1424733

[ref18] Chen T. Sun K.-S. (2012). Exploring the strategy to improve senior citizens’ participations on recreational sports. Knowl.-Based Syst. 26, 86–92. doi: 10.1016/j.knosys.2011.07.008

[ref19] Chishima Y. Mizuno M. Sugawara D. Miyagawa Y. (2018). The influence of self-compassion on cognitive appraisals and coping with stressful events. Mindfulness 9, 1907–1915. doi: 10.1007/s12671-018-0933-0

[ref20] Chwyl C. Chen P. Zaki J. (2020). Beliefs about self-compassion: implications for coping and self-improvement. Personal. Soc. Psychol. Bull. 47, 1327–1342. doi: 10.1177/0146167220965303, PMID: 33166205

[ref21] Clark L. A. Watson D. (2016). Constructing validity: Basic issues in objective scale development. Washington, DC: American Psychological Association.

[ref22] Dahlstrand J. Friberg P. Fridolfsson J. Börjesson M. Arvidsson D. Ekblom Ö. . (2021). The use of coping strategies “shift-persist” mediates associations between physical activity and mental health problems in adolescents: a cross-sectional study. BMC Public Health 21:1104. doi: 10.1186/s12889-021-11158-0, PMID: 34107916 PMC8191033

[ref23] Donnellan C. Hevey D. Hickey A. O’neill D. (2006). Defining and quantifying coping strategies after stroke: a review. J. Neurol. Neurosurg. Psychiatry 77, 1208–1218. doi: 10.1136/jnnp.2005.085670, PMID: 17043290 PMC2077363

[ref24] Doré I. O’loughlin J. L. Beauchamp G. Martineau M. Fournier L. (2016). Volume and social context of physical activity in association with mental health, anxiety and depression among youth. Prev. Med. 91, 344–350. doi: 10.1016/j.ypmed.2016.09.006 27609745

[ref25] Dundas I. Binder P.-E. Hansen T. G. B. Stige S. H. (2017). Does a short self-compassion intervention for students increase healthy self-regulation? A randomized control trial. Scand. J. Psychol. 58, 443–450. doi: 10.1111/sjop.12385, PMID: 28850726

[ref26] Dunne S. Sheffield D. Chilcot J. (2016). Brief report: self-compassion, physical health and the mediating role of health-promoting behaviours. J. Health Psychol. 23, 993–999. doi: 10.1177/1359105316643377, PMID: 27121978

[ref27] Duplaga M. Grysztar M. (2021). The association between future anxiety, health literacy and the perception of the Covid-19 pandemic: A cross-sectional study. Healthcare 9:43. doi: 10.3390/healthcare9010043, PMID: 33466487 PMC7824811

[ref28] Faulkner G. Rhodes R. E. Vanderloo L. M. Chulak-Bozer T. O’reilly N. Ferguson L. . (2020). Physical activity as a coping strategy for mental health due to the Covid-19 virus: a potential disconnect among Canadian adults? Front. Commun. 5:571833. doi: 10.3389/fcomm.2020.571833

[ref29] Ferrari M. Dal Cin M. Steele M. (2017). Self-compassion is associated with optimum self-care behaviour, medical outcomes and psychological well-being in a cross-sectional sample of adults with diabetes. Diabet. Med. 34, 1546–1553. doi: 10.1111/dme.13451, PMID: 28799282

[ref30] Ferretti F. (2015). Unhealthy behaviours: an international comparison. PLoS One 10:e0141834. doi: 10.1371/journal.pone.0141834, PMID: 26512717 PMC4626075

[ref31] Firth J. Solmi M. Wootton R. E. Vancampfort D. Schuch F. B. Hoare E. . (2020). A meta-review of “lifestyle psychiatry”: the role of exercise, smoking, diet and sleep in the prevention and treatment of mental disorders. World Psychiatry 19, 360–380. doi: 10.1002/wps.20773, PMID: 32931092 PMC7491615

[ref32] Floyd D. L. Prentice-Dunn S. Rogers R. W. (2000). A meta-analysis of research on protection motivation theory. J. Appl. Soc. Psychol. 30, 407–429. doi: 10.1111/j.1559-1816.2000.tb02323.x

[ref33] Garbóczy S. Szemán-Nagy A. Ahmad M. S. Harsányi S. Ocsenás D. Rekenyi V. . (2021). Health anxiety, perceived stress, and coping styles in the shadow of the Covid-19. BMC Psychol. 9:53. doi: 10.1186/s40359-021-00560-3, PMID: 33823945 PMC8022303

[ref34] Garcia M.-G. Estrella M. Peñafiel A. Arauz P. G. Martin B. J. (2021). Impact of 10-min daily yoga exercises on physical and mental discomfort of home-office workers during Covid-19. Hum. Factors 65, 1525–1541. doi: 10.1177/00187208211045766, PMID: 34595984 PMC11107138

[ref35] Heinen A. Varghese S. Krayem A. Molodynski A. (2021). Understanding health anxiety in the Covid-19 pandemic. Int. J. Soc. Psychiatry 68, 1756–1763. doi: 10.1177/00207640211057794, PMID: 34823387

[ref36] Ho L. Y. W. (2019). A concept analysis of coping with chronic pain in older adults. Pain Manag. Nurs. 20, 563–571. doi: 10.1016/j.pmn.2019.03.002, PMID: 31103511

[ref37] Howie E. K. Mcveigh J. A. Smith A. J. Zabatiero J. Bucks R. S. Mori T. A. . (2020). Physical activity trajectories from childhood to late adolescence and their implications for health in young adulthood. Prev. Med. 139:106224. doi: 10.1016/j.ypmed.2020.106224, PMID: 32735989

[ref38] Huang L. Kaixin L. Si-Tong C. Yizhen R. Yi Z. And Chi X. (2021). The independent, joint, and additive associations of physical activity and self-compassion on depression symptoms among Chinese college students. Risk Manag. Healthc. Policy. 14, 4673–4683. doi: 10.2147/RMHP.S336709 34824554 PMC8610759

[ref39] Hughes M. Brown S. L. Campbell S. Dandy S. Cherry M. G. (2021). Self-compassion and anxiety and depression in chronic physical illness populations: a systematic review. Mindfulness 12, 1597–1610. doi: 10.1007/s12671-021-01602-y

[ref40] Indregard A.-M. R. Ihlebæk C. M. Eriksen H. R. (2013). Modern health worries, subjective health complaints, health care utilization, and sick leave in the Norwegian working population. Int. J. Behav. Med. 20, 371–377. doi: 10.1007/s12529-012-9246-1, PMID: 22729981

[ref41] Jennings R. E. Lanaj K. Kim Y. J. (2023). Self-compassion at work: a self-regulation perspective on its beneficial effects for work performance and wellbeing. Pers. Psychol. 76, 279–309. doi: 10.1111/peps.12504

[ref42] Jungmann S. M. Witthöft M. (2020). Health anxiety, cyberchondria, and coping in the current Covid-19 pandemic: which factors are related to coronavirus anxiety? J. Anxiety Disord. 73:102239. doi: 10.1016/j.janxdis.2020.102239, PMID: 32502806 PMC7239023

[ref43] Kandola A. Vancampfort D. Herring M. Rebar A. Hallgren M. Firth J. . (2018). Moving to beat anxiety: epidemiology and therapeutic issues with physical activity for anxiety. Curr. Psychiatry Rep. 20:63. doi: 10.1007/s11920-018-0923-x, PMID: 30043270 PMC6061211

[ref44] Kirmizi M. Yalcinkaya G. Sengul Y. S. (2021). Gender differences in health anxiety and musculoskeletal symptoms during the Covid-19 pandemic. J. Back Musculoskelet. Rehabil. 34, 161–167. doi: 10.3233/BMR-200301, PMID: 33682695

[ref45] Koivusilta L. K. Acacio-Claro P. J. Mattila V. M. Rimpelä A. H. (2024). Health and health behaviours in adolescence as predictors of education and socioeconomic status in adulthood – a longitudinal study. BMC Public Health 24:1178. doi: 10.1186/s12889-024-18668-7, PMID: 38671433 PMC11055384

[ref46] Korkmaz S. Kazgan A. Çekiç S. Tartar A. S. Balcı H. N. Atmaca M. (2020). The anxiety levels, quality of sleep and life and problem-solving skills in healthcare workers employed in Covid-19 services. J. Clin. Neurosci. 80, 131–136. doi: 10.1016/j.jocn.2020.07.073, PMID: 33099335 PMC7425768

[ref47] Kotera Y. Van Gordon W. (2021). Effects of self-compassion training on work-related well-being: A systematic review. Front. Psychol. 12, 12–2021. doi: 10.3389/fpsyg.2021.630798, PMID: 33967896 PMC8102699

[ref48] Lathren C. R. Efird-Green L. Reed D. Zimmerman S. Perreira K. M. Bluth K. . (2024). The prevalence and benefits of self-compassion among professional caregivers. J. Am. Med. Dir. Assoc. 25:105099. doi: 10.1016/j.jamda.2024.105099, PMID: 38901466

[ref49] Lebel S. Mutsaers B. Tomei C. Leclair C. S. Jones G. Petricone-Westwood D. . (2020). Health anxiety and illness-related fears across diverse chronic illnesses: A systematic review on conceptualization, measurement, prevalence, course, and correlates. PLoS One 15:e0234124. doi: 10.1371/journal.pone.0234124, PMID: 32716932 PMC7384626

[ref50] Lee L. Petter S. Fayard D. Robinson S. (2011). On the use of partial least squares path modeling in accounting research. Int. J. Account. Inf. Syst. 12, 305–328. doi: 10.1016/j.accinf.2011.05.002

[ref51] Lek Y.-Y. Bishop G. D. (1995). Perceived vulnerability to illness threats: the role of disease type, risk factor perception and attributions. Psychol. Health 10, 205–217. doi: 10.1080/08870449508401950

[ref52] Liang D. (1994). Stress level and its relation with physical activity in higher education. Chin. Ment. Health J. 8, 5–6.

[ref53] Lizano E. L. (2015). Examining the impact of job burnout on the health and well-being of human service workers: a systematic review and synthesis. Hum. Serv. Organ. Manag. Leadersh. Governance 39, 167–181. doi: 10.1080/23303131.2015.1014122

[ref54] Lloyd J. Muers J. Patterson T. G. Marczak M. (2019). Self-compassion, coping strategies, and caregiver burden in caregivers of people with dementia. Clin. Gerontol. 42, 47–59. doi: 10.1080/07317115.2018.1461162 29723129

[ref55] Lu H. Xia S. Zheng Y. Chen W. Jin Z. Sun W. . (2025). The associations between coping resources and help-seeking intention in a sample of Chinese first-year medical students: mediation effects of coping strategies. BMC Public Health 25:1579. doi: 10.1186/s12889-025-22755-8, PMID: 40295998 PMC12036150

[ref56] Macbeth A. Gumley A. (2012). Exploring compassion: A meta-analysis of the association between self-compassion and psychopathology. Clin. Psychol. Rev. 32, 545–552. doi: 10.1016/j.cpr.2012.06.003, PMID: 22796446

[ref57] Mcdowell C. P. Dishman R. K. Gordon B. R. Herring M. P. (2019). Physical activity and anxiety: a systematic review and meta-analysis of prospective cohort studies. Am. J. Prev. Med. 57, 545–556. doi: 10.1016/j.amepre.2019.05.012, PMID: 31542132

[ref58] Meng R. Xiangyu L. Shiming D. Yi L. Dan L. Jing C. . (2020). The mediating role of perceived stress in associations between self-compassion and anxiety and depression: further evidence from Chinese medical workers. Risk Manag. Healthc. Policy 13, 2729–2741. doi: 10.2147/RMHP.S261489, PMID: 33262669 PMC7699983

[ref59] Michalchuk V. F. Lee S.-J. Waters C. M. Hong O. S. Fukuoka Y. (2022). Systematic review of the influence of physical work environment on office workers’ physical activity behavior. Workplace Health Saf. 70, 97–119. doi: 10.1177/21650799211039439, PMID: 35014589 PMC9733787

[ref60] Milne S. Sheeran P. Orbell S. (2000). Prediction and intervention in health-related behavior: a meta-analytic review of protection motivation theory. J. Appl. Soc. Psychol. 30, 106–143. doi: 10.1111/j.1559-1816.2000.tb02308.x

[ref61] Mokhtari R. Moayedi S. Golitaleb M. (2020). Covid-19 pandemic and health anxiety among nurses of intensive care units. Int. J. Ment. Health Nurs. 29, 1275–1277. doi: 10.1111/inm.12800, PMID: 33063915 PMC7675428

[ref62] Monod M. Blenkinsop A. Xi X. Hebert D. Bershan S. Tietze S. . (2021). Age groups that sustain resurging Covid-19 epidemics in the United States. Science 371:eabe8372. doi: 10.1126/science.abe8372 33531384 PMC8101272

[ref63] Moreno-Jiménez J. E. Demerouti E. Blanco-Donoso L. M. Chico-Fernández M. Iglesias-Bouzas M. I. Garrosa E. (2023). Passionate healthcare workers in demanding intensive care units: its relationship with daily exhaustion, secondary traumatic stress, empathy, and self-compassion. Curr. Psychol. 42, 29387–29402. doi: 10.1007/s12144-022-03986-z, PMID: 36406844 PMC9667444

[ref64] Mullet E. Houdbine A. Laumonier S. Girard M. (1998). “Forgivingness”: factor structure in a sample of young, middle-aged, and elderly adults. Eur. Psychol. 3, 289–297. doi: 10.1027/1016-9040.3.4.289

[ref65] Murphy S. L. Kratz A. L. Williams D. A. Geisser M. E. (2012). The association between symptoms, pain coping strategies, and physical activity among people with symptomatic knee and hip osteoarthritis. Front. Psychol. 3:326. doi: 10.3389/fpsyg.2012.00326 22969747 PMC3432514

[ref66] Nagao K. Yoshiike T. Okubo R. Matsui K. Kawamura A. Izuhara M. . (2023). Association between health anxiety dimensions and preventive behaviors during the COVID-19 pandemic among Japanese healthcare workers. Heliyon 9:e22176. doi: 10.1016/j.heliyon.2023.e22176 38034695 PMC10685365

[ref67] Neff K. D. (2011). Self-compassion, self-esteem, and well-being. Soc. Personal. Psychol. Compass 5, 1–12. doi: 10.1111/j.1751-9004.2010.00330.x

[ref68] Neff K. D. (2023). Self-compassion: theory, method, research, and intervention. Annu. Rev. Psychol. 74, 193–218. doi: 10.1146/annurev-psych-032420-031047, PMID: 35961039

[ref69] Neff K. D. Tóth-Király I. Knox M. C. Kuchar A. Davidson O. (2021). The development and validation of the state self-compassion scale (long-and short form). Mindfulness 12, 121–140. doi: 10.1007/s12671-020-01505-4

[ref70] Pasha H. Mahbobeh F. Mohammad C. Fatemeh B. Hemmat G. Somayeh A. . (2022). Health-promotion and health-harming behaviours in pregnant women: role of coping strategies, anxiety, and depression. J. Obstet. Gynecol. 42, 410–415. doi: 10.1080/01443615.2021.1910634 34159886

[ref71] Pastore O. Mcfadden T. Fortier M. (2023). Investigating the impact of physical activity counselling on self-compassion and physical activity. Curr. Psychol. 42, 10951–10963. doi: 10.1007/s12144-021-02346-7

[ref72] Pauley G. Mcpherson S. (2010). The experience and meaning of compassion and self-compassion for individuals with depression or anxiety. Psychol. Psychother. 83, 129–143. doi: 10.1348/147608309X471000, PMID: 19785933

[ref73] Peng R. X. (2022). How online searches fuel health anxiety: investigating the link between health-related searches, health anxiety, and future intention. Comput. Human Behav. 136:107384. doi: 10.1016/j.chb.2022.107384

[ref74] Pereira M. J. Coombes B. K. Comans T. A. Johnston V. (2015). The impact of onsite workplace health-enhancing physical activity interventions on worker productivity: a systematic review. Occup. Environ. Med. 72, 401–412. doi: 10.1136/oemed-2014-102678, PMID: 25780031

[ref75] Phillips W. J. Hine D. W. (2021). Self-compassion, physical health, and health behaviour: a meta-analysis. Health Psychol. Rev. 15, 113–139. doi: 10.1080/17437199.2019.1705872, PMID: 31842689

[ref76] Rebar A. L. Dimmock J. A. Jackson B. Rhodes R. E. Kates A. Starling J. . (2016). A systematic review of the effects of non-conscious regulatory processes in physical activity. Health Psychol. Rev. 10, 395–407. doi: 10.1080/17437199.2016.1183505, PMID: 27118430

[ref77] Rogers R. W. (1975). A protection motivation theory of fear appeals and attitude change1. J. Psychol. 91, 93–114. doi: 10.1080/00223980.1975.9915803, PMID: 28136248

[ref78] Shan B. Liu X. Gu A. Zhao R. (2022). The effect of occupational health risk perception on job satisfaction. Int. J. Environ. Res. Public Health 19:111. doi: 10.3390/ijerph19042111, PMID: 35206297 PMC8872356

[ref79] Shpakou A. Naumau I. A. Krestyaninova T. Y. Znatnova A. V. Lollini S. V. Surkov S. . (2022). Physical activity, life satisfaction, stress perception and coping strategies of university students in Belarus during the Covid-19 pandemic. Int. J. Environ. Res. Public Health 19:629. doi: 10.3390/ijerph19148629, PMID: 35886479 PMC9317606

[ref80] Stubbs B. Koyanagi A. Hallgren M. Firth J. Richards J. Schuch F. . (2017). Physical activity and anxiety: A perspective from the world health survey. J. Affect. Disord. 208, 545–552. doi: 10.1016/j.jad.2016.10.028, PMID: 27802893

[ref81] Sunderland M. Newby J. M. Andrews G. (2013). Health anxiety in Australia: prevalence, comorbidity, disability and service use. Br. J. Psychiatry 202, 56–61. doi: 10.1192/bjp.bp.111.103960, PMID: 22500013

[ref82] Taylor H. Cavanagh K. Field A. P. Strauss C. (2022). Health care workers’ need for headspace: findings from a multisite definitive randomized controlled trial of an unguided digital mindfulness-based self-help app to reduce healthcare worker stress. JMIR Mhealth Uhealth 10:e31744. doi: 10.2196/31744, PMID: 36006668 PMC9459942

[ref83] Teasdale E. Yardley L. Schlotz W. Michie S. (2012). The importance of coping appraisal in behavioural responses to pandemic flu. Br. J. Health Psychol. 17, 44–59. doi: 10.1111/j.2044-8287.2011.02017.x, PMID: 22233104

[ref84] Terry M. L. Leary M. R. Mehta S. Henderson K. (2013). Self-compassionate reactions to health threats. Personal. Soc. Psychol. Bull. 39, 911–926. doi: 10.1177/0146167213488213, PMID: 23813424

[ref85] Tobin D. L. Holroyd K. A. Reynolds R. V. Wigal J. K. (1989). The hierarchical factor structure of the coping strategies inventory. Cognit. Ther. Res. 13, 343–361. doi: 10.1007/BF01173478

[ref86] Tyrer P. (2018). Recent advances in the understanding and treatment of health anxiety. Curr. Psychiatry Rep. 20:49. doi: 10.1007/s11920-018-0912-0, PMID: 29931576

[ref87] Vagni M. Maiorano T. Giostra V. Pajardi D. Bartone P. (2022). Emergency stress, hardiness, coping strategies and burnout in health care and emergency response workers during the Covid-19 pandemic. Front. Psychol. 13:918788. doi: 10.3389/fpsyg.2022.918788, PMID: 35800954 PMC9253608

[ref88] Van Dam N. T. Sheppard S. C. Forsyth J. P. Earleywine M. (2011). Self-compassion is a better predictor than mindfulness of symptom severity and quality of life in mixed anxiety and depression. J. Anxiety Disord. 25, 123–130. doi: 10.1016/j.janxdis.2010.08.011, PMID: 20832990

[ref89] Vintila M. Tudorel O. I. Stefanut A. Ivanoff A. Bucur V. (2023). Emotional distress and coping strategies in Covid-19 anxiety. Curr. Psychol. 42, 17503–17512. doi: 10.1007/s12144-021-02690-8, PMID: 35035193 PMC8744025

[ref90] Vos L. M. W. Habibović M. Nyklíček I. Smeets T. Mertens G. (2021). Optimism, mindfulness, and resilience as potential protective factors for the mental health consequences of fear of the coronavirus. Psychiatry Res. 300:113927. doi: 10.1016/j.psychres.2021.113927, PMID: 33848964 PMC9755114

[ref91] Wagner R. W. Mallaiah S. Anderson C. R. Engle R. Vasu V. Bruera E. . (2024). Effects of the brief Simha Kriya breathing practice for health care workers during the Covid-19 pandemic. J. Integr. Complement. Med. 30, 970–977. doi: 10.1089/jicm.2023.0692, PMID: 38546421

[ref92] Wandel M. Roos G. (2005). Work, food and physical activity. A qualitative study of coping strategies among men in three occupations. Appetite 44, 93–102. doi: 10.1016/j.appet.2004.08.002, PMID: 15604036

[ref93] Wang Y. Fu T. Wang J. Chen S. Sun G. (2024). The relationship between self-compassion, coping style, sleep quality, and depression among college students. Front. Psychol. 15:1378181. doi: 10.3389/fpsyg.2024.1378181, PMID: 38911963 PMC11190380

[ref94] Wang Z. Li C. Xie Z. Hong O. (2024). Grit difference in the association between academic stress and adolescents’ meaning in life: the roles of school burnout and self-compassion. Child Care Health Dev. 50:e70005. doi: 10.1111/cch.70005, PMID: 39540694

[ref95] Wang K. Li Y. Zhang T. Luo J. (2022). The relationship among college students’ physical exercise, self-efficacy, emotional intelligence, and subjective well-being. Int. J. Environ. Res. Public Health 19:596. doi: 10.3390/ijerph191811596, PMID: 36141869 PMC9517190

[ref96] Wang H. Zhang Q. Lin Y. Liu Y. Xu Z. Yang J. (2023). Keep moving to retain the healthy self: the influence of physical exercise in health anxiety among Chinese menopausal women. Behav. Sci. 13:140. doi: 10.3390/bs13020140, PMID: 36829369 PMC9952320

[ref97] Wechsler B. (1995). Coping and coping strategies: a behavioural view. Appl. Anim. Behav. Sci. 43, 123–134. doi: 10.1016/0168-1591(95)00557-9

[ref98] Wen Y. Yang Y. Shen J. Luo S. (2021). Anxiety and prognosis of patients with myocardial infarction: A meta-analysis. Clin. Cardiol. 44, 761–770. doi: 10.1002/clc.23605, PMID: 33960435 PMC8207975

[ref99] Witte K. (1992). Putting the fear back into fear appeals: the extended parallel process model. Commun. Monogr. 59, 329–349. doi: 10.1080/03637759209376276

[ref100] Yang X. Ilias K. Anuar Md Isa K. Li Q.-H. Wang H.-B. Li H. (2025). The role of healthy personality, psychological flexibility, and coping mechanisms in university students’ mental health in China. Front. Psychol. 16:1578793. doi: 10.3389/fpsyg.2025.1578793 40823425 PMC12355609

[ref101] Yu X. Xing S. Yang Y. (2025). The relationship between psychological capital and athlete burnout: the mediating relationship of coping strategies and the moderating relationship of perceived stress. Bmc Psychol. 13:64. doi: 10.1186/s40359-025-02379-8, PMID: 39849608 PMC11756217

[ref102] Zhang Y. (2022). Structural barriers and narratives of Chinese social workers’ coping strategies. Hum. Serv. Organ. Manag. Leadersh. Gov. 46, 370–391. doi: 10.1080/23303131.2022.2046669

[ref103] Zhang S. Pringle A. Roscoe C. (2025). Self-compassion improves barrier self-efficacy and subsequently physical activity: A test of longitudinal mediation using a representative sample of the United Kingdom. Br. J. Health Psychol. 30:e12757. doi: 10.1111/bjhp.12757, PMID: 39424408 PMC11586801

[ref104] Zhang S. Roscoe C. Pringle A. (2023). Self-compassion and physical activity: the underpinning role of psychological distress and barrier self-efficacy. Int. J. Environ. Res. Public Health 20:480. doi: 10.3390/ijerph20021480, PMID: 36674235 PMC9859314

[ref105] Zheng C. Kia K. Di F. John M. And Ee M. S. (2016). Impact of individual coping strategies and organisational work–life balance programmes on Australian employee well-being. Int. J. Hum. Resour. Manag. 27, 501–526. doi: 10.1080/09585192.2015.1020447

